# Acquired hemophilia A in a woman with systemic lupus erythematosus: A case report and review of literature

**DOI:** 10.1097/MD.0000000000022926

**Published:** 2020-10-23

**Authors:** Pan Shen, Jing Li, Shenghao Tu, Gang Chen, Chao Chen

**Affiliations:** a Department of Integrated Traditional Chinese and Western Medicine, Tongji Hospital; b Department of Orthopaedics, Union Hospital, Tongji Medical College, Huazhong University of Science and Technology, Wuhan, China.

**Keywords:** acquired hemophilia A, factor VIII inhibitor, hematuria, systemic lupus erythematosus

## Abstract

**Rationale:**

Acquired hemophilia A (AHA) is a rare autoimmune disease caused by autoantibodies directed against the activity of factor VIII (FVIII) and presents with prolonged bleeding. 5.7% of systemic lupus erythematosus (SLE) patients are affected by AHA.

**Patient concerns:**

A 51-year-old female patient with SLE presenting with the fatigue and spontaneous clinical bleeding symptoms such as hematuria and ecchymoses for 1 week.

**Diagnosis:**

Laboratory examinations revealed prolongation of the activated partial thromboplastin time (APTT) (65.7 s), decreased FVIII activity (1.4%), and a titer of FVIII inhibitors of 8.5 Bethesda units/mL.

**Interventions:**

Transfusion of recombinant human FVIII (ADVATE) in combination with intravenous methylprednisolone, cyclophosphamide, plasmapheresis, and fresh frozen plasma successfully stopped the bleeding and reduced the level of FVIII inhibitor.

**Outcomes:**

The size of the hematoma slowly decreased. The skin ecchymosis was gradually absorbed, the hemoglobin count increased, and the coagulation index gradually improved. There was no new bleeding or bleeding site. The patient was discharged and transferred to a local hospital for hospice care.

**Lessons:**

AHA in a patient with SLE is rare. Once it occurs, it can be life-threatening. Clinicians should remain aware that because some cases of AHA may have features of SLE, appropriate distinction and diagnosis of these different but associated diseases is necessary.

## 1 Introduction

Systemic lupus erythematosus (SLE) is an autoimmune disease that can affect various organs and systems including joints, cardiovascular system, lung, skin, kidneys, nervous system, and blood.^[1]^ Acquired hemophilia A (AHA) is a rare autoimmune disorder resulting from autoantibodies against the activity of clotting factor VIII (FVIII) and presents with prolonged bleeding,^[2]^ with an annual incidence rate of 1 to 1.5 in 1,000,000.^[3]^ It has been reported that autoimmune diseases, pregnancy, drug reactions, and malignancy can induce AHA.^[4]^ The hemorrhagic syndrome is rapid, can occur without a history of coagulopathy, and can be suddenly invoked in the presence of autoimmunity. Severe bleeding may occur in ∼80% of patients. Because of its high mortality rate, the treatment of these patients can be a clinical challenge.^[5]^ Initially, treatment^[6]^ of AHA includes the control of bleeding and its complications by desmopressin, recombinant or plasma-derived human FVIII, porcine FVIII, activated prothrombin complex concentrates (aPCC), reduction of the titer of FVIII inhibitors^[7]^ and elimination of the autoantibodies with corticosteroids, intravenous immunoglobulins, rituximab, and immunosuppressive and immunomodulating agents (cyclophosphamide, azathioprine, and cyclosporine).

In this report, we present the life-threatening case of a 51-year-old woman with SLE who developed hemorrhagic syndrome caused by AHA owing to the existence of FVIII inhibitors. Combination therapy with cyclophosphamide (CTX), methylprednisolone, fresh plasma, plasmapheresis, and recombinant human FVIII was effective in regulating and reducing the level of FVIII inhibitor, and successfully resolved her life-threatening condition.

## 2 Method

As this study is a case report, Ethical approval was waived by the Ethical Committee of Tongji hospital, Tongji Medical College, Huazhong University of Science and Technology. Written informed consent was obtained from the patient for publication of this case report and accompanying images.

## 3 Case presentation

A 51-year-old Chinese woman consulted a hospital because of multiple erythema, fatigue, polyarthralgia, and thrombocytopenia in May 2019. She was diagnosed with SLE, based on positive results of anti-nuclear antibodies (ANA), anti-SS-A/Ro antibodies, a hematological disorder, proteinuria, and arthritis. At that time, coagulation test results were not within normal ranges (hypoplatelets). She received oral treatment with prednisone starting at 45 mg/day with a gradually decreasing dose. In July 2019, she was taken to the emergency department with fatigue and spontaneous subcutaneous bleeding of 1 week's duration. Laboratory chemistry tests revealed a positive ANA (1:100 titer) and anti-SS-A/Ro (207.87 RU/mL). The platelet level had decreased to 55 × 10^9^/L. On August 1, 2019 she was admitted for further treatment.

Physical examination results on admission revealed obvious subcutaneous ecchymoses in the limbs and left lower abdomen as well as double lower-extremity edema. She denied any history of hematologic disorder or family history of bleeding disease. The laboratory results on 2th August were as follows: hemoglobin, 55 g/L; white blood cell count, 12.01 × 10^9^/L; platelet count, 37.0 × 10^9^/L; blood albumin, 32.1 g/L; C3, 0.37 g/L; C4, 0.07 g/L; triglycerides, 2.35 mmol/L; creatine kinase, 232 U/L; lactate dehydrogenase, 365 U/L; sodium, 134.7 mmol/L; calcium, 2.10 mmol/L; phosphorus, 0.76 mmol/L; potassium, 3.25 mmol/L; C-reactive protein, 8.8 mg/L; urinary red blood cell count, >20,000/μL; urine protein, +++; prothrombin time, 15.1 s; APTT, 65.7 s; D-D dimer quantification, 2.60 FEU.

Color Doppler ultrasonography revealed left lower extremity hematoma, pericardial effusion, ascites, and pelvic fluid. Further sonography of her right jugular vein showed evidence of thrombosis.

Combining these findings with pre-hospital tests, we confirmed the diagnosis of SLE and thrombocytopenia, with SLE in an active state. The patient was given prednisone 40 mg once a day orally. However, after treatment with red blood cells, recombinant human erythropoietin, platelets, fresh plasma, aminotoluic acid, phenethylamine, and vitamin K1, her coagulation function and subcutaneous ecchymosis did not improve, while APTT intermittently increased and reached a maximum of 97.7 s on the 8th hospital day. Disease progression was not stopped by the treatment.

On 13th August, the quantitative detection of FVIII activity was detected, the test result was 2% (normal 50%–150%), the activity of coagulation factor XI was normal, and the FVIII inhibitor level was 8.5 Bethesda units (BU)/mL, leading us to speculate that the patient was suffering from SLE merged with AHA. She was administered methylprednisolone intravenously 80 mg once a day, CTX 200 mg intravenously once a week, and recombinant human FVIII 250 IU once daily, along with supplemental plasma and plasmapheresis to relieve bleeding and disease activity. The skin ecchymosis was gradually absorbed, the hemoglobin count increased, and the coagulation index gradually improved after 17 days of administration of FVIII. (Until August 31, APTT decreased from 97.7 to 39.7 s; FVIII increased from 2% to 56.6%; FVIII inhibitor decreased from 8.5 to 1.02 BU/mL). There was no new bleeding or bleeding site, and the methylprednisolone dose was gradually reduced. The size of the hematoma slowly decreased. The patient's overall condition improved and she was discharged home on 11th September (FVIII 97.0%, FVIII inhibitor 0.62 BU/mL). After discharge, the patient received prednisone 40 mg/day orally treatment, and gradually reduce the dose to the maintenance level. She has received intravenously administered CTX 200 mg/week for 1 month. After that, CTX was used every 2 weeks for 4 months. The patient has been followed up in the outpatient clinic so far, she is still in complete remission (FVIII activity 121%).

## 4 Discussion

SLE is a chronic autoimmune disease characterized by the formation of autoantibodies and inflammation in multiple organs. The clinical manifestations of SLE are heterogeneous, so the diagnosis can be challenging.^[8]^ Therapeutic strategies mainly involve immunoregulation and immunosuppression with the aim of relieving the specific organ manifestations and achieving low disease activity.^[9]^ AHA is a severe life-threatening autoimmune disease resulting from autoantibodies directed against coagulation factors, the most common being FVIII. The mortality rate of AHA has been reported to be approximately in the range of 8%–20%.^[10]^ If autoantibodies are not removed in time, fatal bleeding and death may occur up to 5 months after the first symptoms appear. AHA is usually associated with collagen vascular diseases, such SLE, rheumatoid arthritis, and dermatomyositis, although no underlying disorders or conditions have been found in approximately 50% of cases.^[11]^ According to reports, 5.7% of SLE patients are also affected by AHA.^[12]^ The mechanism of AHA in SLE patients is not yet fully understood. It may be related to the production of FVIII antibodies or vWF factor autoantibodies in SLE patients. The occurrence of AHA is caused by the formation of circulating immune complexes with FVIII when specific or non-specific autoantibodies are formed, which are then eliminated by cells carrying the corresponding Fc segment receptors. Most of these autoantibodies belong to the IgG4 subgroup and bind to the area C2, A2, or A3 of FVIII. It is believed that the bleeding of AHA patients secondary to SLE is caused by the binding of non-specific autoantibodies to vWF factor or plasma FVIII and vWF complex to form an immune complex, which in turn binds to the Fc receptor. The nuclear-macrophage system is eliminated, resulting in a decrease in the quantity and quality of plasma vWF factors. In addition, in the presence of autoantibodies, the formation of vWF factor polymer multimers may also be related to the occurrence of AHA.

The diagnosis is based on prolonged APTT, reduction of FVIII level, and presence of an FVIII inhibitor.^[13]^ The level of FVIII inhibitor is detected by using the Bethesda assay or a mixing test in a patient without preceding individual or family history of hemorrhagic syndrome. The bleeding features of AHA are different from those of congenital hemophilia A.^[14]^ In most cases of AHA, including the current patient, FVIII inhibitors lead to hemorrhage in the skin and mucous membranes, gastrointestinal bleeding, urinary tract bleeding, retroperitoneal bleeding, whereas hemarthrosis, a predominant characteristic of congenital FVIII defect, is not common. Congenital hemophilia A is an X chromosome-linked single-gene recessive hereditary bleeding disorder. The lack of FVIII is caused by mutations in the corresponding clotting factor gene. Congenital hemophilia A coagulation abnormality is mainly manifested in clinically as repeated bleeding since childhood. The bleeding site is mainly in the deep tissues of muscles and joints where endogenous coagulation mechanisms dominate. In severe cases, even internal organ bleeding. In addition, it is often difficult for hemophilia patients to hemorrhage from wounds after trauma or bleeding after hemostasis.

In the present case, our patient with SLE and AHA had no previous history of bleeding and no family history of hemophilia or delayed bleeding after medical procedures, while coagulation showed prolonged APTT. Further examination revealed a decrease in FVIII and a significant increase in FVIII inhibitor concentration, and bleeding occurred during the active period of SLE, leading us to a clear diagnosis of AHA. When APTT is in an abnormal range, combined tests should be conducted to determine between the presence of inhibitors and factor deficiency. If the prolonged APTT is corrected, this often suggests a coagulation factor defect; otherwise it is likely to be presence of inhibitor against the clotting factor.

We used PubMed, Scopus, and ScienceDirect medical search engines to complete the review of literature, using the following keyword search terms: “lupus and acquired haemophilia”; “systemic lupus erythematosus and acquired haemophilia”; “systemic lupus erythematosus and factor VIII inhibitor”; “lupus and factor VIII inhibitor.” Among preceding studies of AHA combined with SLE, we acquired 9 full-text articles.^[15–23]^ The main characteristics of the previously reported cases are listed in Table 1. These cases involve female and male patients with a median age of 46.7 years (range, 24–69 years). The patients with quiescent SLE also had inhibitory autoantibodies, indicating that the existence of FVIII inhibitor and its concentration is not always related to potential disease activity. Furthermore, level of FVIII inhibitor does not regularly correlate with the severity of hemorrhagic symptoms. One possible reason for this is that the inhibitory autoantibodies do not fully function to neutralize the activity of FVIII. We were unable to ascertain other potential factors for our SLE patient who developed AHA, such as infections, use of certain drugs, or other disease conditions. On examining the reported cases, treatment with immunoglobulin, glucocorticoids (GC), cyclosporine A, and anti-CD20 monoclonal antibody appears to represent the prevailing therapy for SLE patients with AHA.

**Table 1:**
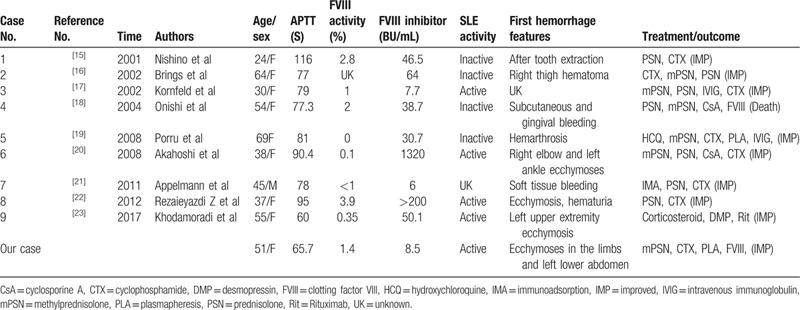
Review of reported cases with AHA and SLE in patients.

Therapeutic strategies consist of anti-hemorrhagic treatment and prohibition of FVIII inhibitor.^[24]^ Treatment management to suppress bleeding involves fresh plasma, desmopressin, FVIII concentrates, aPCC, and recombinant human or porcine FVIII. It is also crucial to recommend an appropriate long-term immunosuppressive approach to eliminate FVIII inhibitor and achieve complete remission. The most commonly used agents include disease-modifying anti-rheumatics, cytotoxic drugs, corticosteroids including cyclosporine A and CTX, or combination treatment, as well as plasmapheresis or immunoadsorption. Recently, rituximab (an anti-CD20 monoclonal antibody) has been applied to the therapy for AHA, with positive results.^[25]^ The sustained reaction is considered to be retained if FVIII inhibitor is less than 0.6 BU/mL and the activity of FVIII is greater than 50%, and if partial remission is established by an inhibitor titer of less than 5 BU/mL and FVIII activity of 30%.

Desmopressin, a useful option for minor bleeding, can promote the release of FVIII, increase the level of plasma von Willebrand factor, and release tissue-type plasminogen activator. Studies have shown that desmopressin can significantly shorten the bleeding time and APTT of patients, with the FVIII increasing by 20% to 60% after administration, though not for severe bleeding. Patients with active serious bleeding with a low level of inhibitor (≤5 BU/mL) may be administered recombinant FVIII, factor VIIa, or aPCC. Human FVIII concentration as a first-line strategy and has proved efficient in some patients.^[26]^ However, human recombinant FVIII is not effective in the presence of high titer inhibitors, unlike other aforementioned factors. In the case of refractory bleeding, plasmapheresis or an immunosorbent assay can be used to quickly remove inhibitors and achieve effective hemostasis. Destruction of inhibitor demands the management of immunosuppressors, although there are no plausible data to suggest the superiority of one agent over another. Most commonly applied schemes include GC alone, GC plus CTX, and GC plus rituximab.^[27]^ Azathioprine, vincristine, mycophenolate mofetil, and cyclosporine can be also used if these treatment regimens are not effective. We recommend also that AHA should be added to the hematological diagnostic criteria for SLE.

Regarding the present patient, after a diagnosis of AHA a large dose of GC was actively administered and CTX-induced remission treatment and plasmapheresis was added. The FVIII was monitored, the FVIII inhibitor titer significantly decreased, and the patient's condition improved. Because FVIII inhibitor levels do not predict the risk of bleeding, they cannot be used to determine whether immunosuppressive therapy should be used, but can predict the patient's response to immunosuppressive therapy. This patient had a relatively high FVIII inhibitor titer and an intensive infusion. After CTX and plasma exchange, the clinical response was better and the laboratory indicators gradually recovered. A serious potential problem in a patient with AHA is the anticipation of massive fatal hemorrhage. For this reason our patient was given recombinant human FVIII, a pro-hemostatic agent originally designed for the treatment of alloimmune hemophiliacs, as well as CTX to better restrain the inflammatory reactivation of SLE.

AHA is so rare that it is clinically easy to miss the diagnosis or to misdiagnose the condition. Once it occurs, it can be life-threatening and requires rapid diagnosis.^[28]^ FVIII inhibitors are most common in autoimmune diseases, and clinicians must ensure that the coagulation results are not abnormal before performing invasive procedures. In autoimmune or rheumatic diseases, when patients present with spontaneous hemorrhage and delayed bleeding combined with prolonged APTT, AHA should be suspected and a complete laboratory assessment is critical, including detection of coagulation, FVIII, and FVIII inhibitors.^[29]^ Once AHA is diagnosed and treated urgently, immunosuppressive therapy is important in addition to supplementation and recovery of hemostasis. There are currently no standard treatment options for SLE combined with AHA, although GC plus immunosuppressants remains the primary approach.^[30]^

Our case emphasizes the significance of timely diagnosis and effective therapy (GC, plasmapheresis, recombinant human FVIII, and CTX) in acquiring a positive result. Further research to decide on the optimal treatment for SLE combined with AHA, according to the severity of the disease and the titer of the inhibitor, is warranted. Combination therapy for SLE patients with AHA should open up opportunities to treat coexisting autoimmune diseases via a multidisciplinary strategy.

## Author contributions

**Funding acquisition:** Chao Chen, Jing Li, Gang Chen.

**Investigation:** Pan Shen.

**Methodology:** Jing Li.

**Resources:** Shenghao Tu.

**Software:** Gang Chen.

**Supervision:** Chao Chen.

**Writing – original draft:** Pan Shen.

**Writing – review & editing:** Pan Shen.
